# Supplementary material for: The effects of genital myiasis on the diversity of the vaginal microbiota in female Bactrian camels
Source: BMC Vet Res. 2022 Mar 5;18:87. doi: 10.1186/s12917-022-03189-5 (PMC8897907; doi:10.1186/s12917-022-03189-5)
Supplement: Supplementary file 5 — Additional file 5. [file 12917_2022_3189_MOESM5_ESM.zip › MPL201709200_16s_yy/Treat1/css/js_css/pdfmake-master/examples/pdfs/lists.pdf]

Unordered list

1. item 1
2. item 2
3. item 3

Unordered list with longer lines

1. item 1
2. Lorem ipsum dolor sit amet, consectetur adipisicing elit. Malit profecta versatur nomine ocurreret multavit
3. item 3

Nested lists

1. item 1
2. Lorem ipsum dolor sit amet, consectetur adipisicing elit. Malit profecta versatur nomine ocurreret multavit
    1. subitem 1
    2. subitem 2
    3. subitem 3 - Lorem ipsum dolor sit amet, consectetur adipisicing elit. Malit profecta versatur nomine ocurreret multavit
    4. subitem 3 - Lorem ipsum dolor sit amet, consectetur adipisicing elit. Malit profecta versatur nomine ocurreret multavit
    5. subitem 3 - Lorem ipsum dolor sit amet, consectetur adipisicing elit. Malit profecta versatur nomine ocurreret multavit
    6. subitem 3 - Lorem ipsum dolor sit amet, consectetur adipisicing elit. Malit profecta versatur nomine ocurreret multavitsubitem 3 - Lorem ipsum dolor sit amet, consectetur adipisicing elit. Malit profecta versatur nomine ocurreret multavitsubitem 3 - Lorem ipsum dolor sit amet, consectetur adipisicing elit. Malit profecta versatur nomine ocurreret multavitsubitem 3 - Lorem ipsum dolor sit amet, consectetur adipisicing elit. Malit profecta versatur nomine ocurreret multavitsubitem 3 - Lorem ipsum dolor sit amet, consectetur adipisicing elit. Malit profecta versatur nomine ocurreret multavitsubitem 3 - Lorem ipsum dolor sit amet, consectetur adipisicing elit. Malit profecta versatur nomine ocurreret multavitsubitem 3 - Lorem ipsum dolor sit amet, consectetur adipisicing elit. Malit profecta versatur nomine ocurreret multavitsubitem 3 - Lorem ipsum dolor sit amet, consectetur adipisicing elit. Malit profecta versatur nomine ocurreret multavit
    7. subitem 3 - Lorem ipsum dolor sit amet, consectetur adipisicing elit. Malit profecta versatur nomine ocurreret multavit
    8. subitem 3 - Lorem ipsum dolor sit amet, consectetur adipisicing elit. Malit profecta versatur nomine ocurreret multavit
    9. subitem 3 - Lorem ipsum dolor sit amet, consectetur adipisicing elit. Malit profecta versatur nomine ocurreret multavit
    10. subitem 3 - Lorem ipsum dolor sit amet, consectetur adipisicing elit. Malit profecta versatur nomine ocurreret multavit
    11. subitem 4
    12. subitem 5

4. item 3
   second line of item3

Lists inside columns

- item 1
- Lorem ipsum dolor sit amet, consectetur adipisicing elit. Malit profecta versatur nomine ocurreret multavit

- item 1
- Lorem ipsum dolor sit amet, consectetur adipisicing elit. Malit profecta versatur nomine ocurreret multavit

Unordered list

- item 1
- item 2
- item 3

Unordered list with longer lines

- item 1
- Lorem ipsum dolor sit amet, consectetur adipisicing elit. Malit profecta versatur nomine ocurreret multavit
- item 3

Nested lists

1. item 1
2. Lorem ipsum dolor sit amet, consectetur adipisicing elit. Malit profecta versatur nomine ocurreret multavit
   - subitem 1
   - subitem 2
   - subitem 3 - Lorem ipsum dolor sit amet, consectetur adipisicing elit. Malit profecta versatur nomine ocurreret multavit
   - subitem 3 - Lorem ipsum dolor sit amet, consectetur adipisicing elit. Malit profecta versatur nomine ocurreret multavit
   - subitem 3 - Lorem ipsum dolor sit amet, consectetur adipisicing elit. Malit profecta versatur nomine ocurreret multavit
   - subitem 3 - Lorem ipsum dolor sit amet, consectetur adipisicing elit. Malit profecta versatur nomine ocurreret multavitsubitem 3 - Lorem ipsum dolor sit amet, consectetur adipisicing elit. Malit profecta versatur nomine ocurreret multavitsubitem 3 - Lorem ipsum dolor sit amet, consectetur adipisicing elit. Malit profecta versatur nomine ocurreret multavitsubitem 3 - Lorem ipsum dolor sit amet, consectetur adipisicing elit. Malit profecta versatur nomine ocurreret multavitsubitem 3 - Lorem ipsum dolor sit amet, consectetur adipisicing elit. Malit profecta versatur nomine ocurreret multavitsubitem 3 - Lorem ipsum dolor sit amet, consectetur adipisicing elit. Malit profecta versatur nomine ocurreret multavitsubitem 3 - Lorem ipsum dolor sit amet, consectetur adipisicing elit. Malit profecta versatur nomine ocurreret multavitsubitem 3 - Lorem ipsum dolor sit amet, consectetur adipisicing elit. Malit profecta versatur nomine ocurreret multavit

    - subitem 3 - Lorem ipsum dolor sit amet, consectetur adipisicing elit. Malit profecta versatur nomine ocurreret multavit
    - subitem 3 - Lorem ipsum dolor sit amet, consectetur adipisicing elit. Malit profecta versatur nomine ocurreret multavit
    - subitem 3 - Lorem ipsum dolor sit amet, consectetur adipisicing elit. Malit profecta versatur nomine ocurreret multavit
    - subitem 3 - Lorem ipsum dolor sit amet, consectetur adipisicing elit. Malit profecta versatur nomine ocurreret multavit
    - subitem 4
    - subitem 5
4. item 3
   second line of item3

Lists inside columns

- item 1
- Lorem ipsum dolor sit amet, consectetur adipisicing elit. Malit profecta versatur nomine ocurreret multavit

- item 1
- Lorem ipsum dolor sit amet, consectetur adipisicing elit. Malit profecta versatur nomine ocurreret multavit

Unordered list

- item 1
- item 2
- item 3

Unordered list with longer lines

- item 1
- Lorem ipsum dolor sit amet, consectetur adipisicing elit. Malit profecta versatur nomine ocurreret multavit
- item 3

Nested lists

- item 1
- Lorem ipsum dolor sit amet, consectetur adipisicing elit. Malit profecta versatur nomine ocurreret multavit
  1. column 1 column 2 column 3 column 4
     - item 1
     - item 2
       - item
       - item
       - item
     - item 4

     subitem 1 in a vertical container
     subitem 2 in a vertical container

2. subitem 2
3. subitem 3 - Lorem ipsum dolor sit amet, consectetur adipisicing elit. Malit profecta versatur nomine ocurreret multavit
4. subitem 3 - Lorem ipsum dolor sit amet, consectetur adipisicing elit. Malit profecta versatur nomine ocurreret multavit
5. subitem 3 - Lorem ipsum dolor sit amet, consectetur adipisicing elit. Malit profecta versatur nomine ocurreret multavit
6. subitem 3 - Lorem ipsum dolor sit amet, consectetur adipisicing elit. Malit profecta versatur nomine ocurreret multavitsubitem 3 - Lorem ipsum dolor sit amet, consectetur adipisicing elit. Malit profecta versatur nomine ocurreret multavitsubitem 3 - Lorem ipsum dolor sit amet, consectetur adipisicing elit. Malit profecta versatur nomine ocurreret multavitsubitem 3 - Lorem ipsum dolor sit amet, consectetur adipisicing elit. Malit profecta versatur nomine ocurreret multavitsubitem 3 - Lorem ipsum dolor sit amet, consectetur adipisicing elit. Malit profecta versatur nomine ocurreret multavitsubitem 3 - Lorem ipsum dolor sit amet, consectetur adipisicing elit. Malit profecta versatur nomine ocurreret multavitsubitem 3 - Lorem ipsum dolor sit amet, consectetur adipisicing elit. Malit profecta versatur nomine ocurreret multavitsubitem 3 - Lorem ipsum dolor sit amet, consectetur adipisicing elit. Malit profecta versatur nomine ocurreret multavit
7. subitem 3 - Lorem ipsum dolor sit amet, consectetur adipisicing elit. Malit profecta versatur nomine ocurreret multavit
8. subitem 3 - Lorem ipsum dolor sit amet, consectetur adipisicing elit. Malit profecta versatur nomine ocurreret multavit
9. subitem 3 - Lorem ipsum dolor sit amet, consectetur adipisicing elit. Malit profecta versatur nomine ocurreret multavit
10. subitem 3 - Lorem ipsum dolor sit amet, consectetur adipisicing elit. Malit profecta versatur nomine ocurreret multavit
11. subitem 4
12. subitem 5

- item 3
  second line of item3

Lists inside columns

- item 1
- Lorem ipsum dolor sit amet, consectetur adipisicing elit. Malit profecta versatur nomine ocurreret multavit

- item 1
- Lorem ipsum dolor sit amet, consectetur adipisicing elit. Malit profecta versatur nomine ocurreret multavit

Unordered list

- item 1
- item 2
- item 3

Unordered list with longer lines

- item 1
- Lorem ipsum dolor sit amet, consectetur adipisicing elit. Malit profecta versatur nomine

ocurreret multavit
- item 3

Nested lists

- item 1
- Lorem ipsum dolor sit amet, consectetur adipisicing elit. Malit profecta versatur nomine ocurreret multavit
  - subitem 1
  - subitem 2
  - subitem 3 - Lorem ipsum dolor sit amet, consectetur adipisicing elit. Malit profecta versatur nomine ocurreret multavit
  - subitem 3 - Lorem ipsum dolor sit amet, consectetur adipisicing elit. Malit profecta versatur nomine ocurreret multavit
  - subitem 3 - Lorem ipsum dolor sit amet, consectetur adipisicing elit. Malit profecta versatur nomine ocurreret multavit
  - subitem 3 - Lorem ipsum dolor sit amet, consectetur adipisicing elit. Malit profecta versatur nomine ocurreret multavitsubitem 3 - Lorem ipsum dolor sit amet, consectetur adipisicing elit. Malit profecta versatur nomine ocurreret multavitsubitem 3 - Lorem ipsum dolor sit amet, consectetur adipisicing elit. Malit profecta versatur nomine ocurreret multavitsubitem 3 - Lorem ipsum dolor sit amet, consectetur adipisicing elit. Malit profecta versatur nomine ocurreret multavitsubitem 3 - Lorem ipsum dolor sit amet, consectetur adipisicing elit. Malit profecta versatur nomine ocurreret multavitsubitem 3 - Lorem ipsum dolor sit amet, consectetur adipisicing elit. Malit profecta versatur nomine ocurreret multavitsubitem 3 - Lorem ipsum dolor sit amet, consectetur adipisicing elit. Malit profecta versatur nomine ocurreret multavitsubitem 3 - Lorem ipsum dolor sit amet, consectetur adipisicing elit. Malit profecta versatur nomine ocurreret multavit
  - subitem 3 - Lorem ipsum dolor sit amet, consectetur adipisicing elit. Malit profecta versatur nomine ocurreret multavit
  - subitem 3 - Lorem ipsum dolor sit amet, consectetur adipisicing elit. Malit profecta versatur nomine ocurreret multavit
  - subitem 3 - Lorem ipsum dolor sit amet, consectetur adipisicing elit. Malit profecta versatur nomine ocurreret multavit
  - subitem 3 - Lorem ipsum dolor sit amet, consectetur adipisicing elit. Malit profecta versatur nomine ocurreret multavit
  - subitem 4
  - subitem 5
- item 3
  second line of item3

Lists inside columns

- item 1
- Lorem ipsum dolor sit amet, consectetur adipisicing elit. Malit profecta versatur nomine ocurreret multavit

- item 1
- Lorem ipsum dolor sit amet, consectetur adipisicing elit. Malit profecta versatur nomine ocurreret multavit

Unordered list

- item 1
- item 2
- item 3

Unordered list with longer lines

- item 1
- Lorem ipsum dolor sit amet, consectetur adipisicing elit. Malit profecta versatur nomine ocurreret multavit
- item 3

Nested lists

- item 1
- Lorem ipsum dolor sit amet, consectetur adipisicing elit. Malit profecta versatur nomine ocurreret multavit
  - subitem 1
  - subitem 2
  - subitem 3 - Lorem ipsum dolor sit amet, consectetur adipisicing elit. Malit profecta versatur nomine ocurreret multavit
  - subitem 3 - Lorem ipsum dolor sit amet, consectetur adipisicing elit. Malit profecta versatur nomine ocurreret multavit
  - subitem 3 - Lorem ipsum dolor sit amet, consectetur adipisicing elit. Malit profecta versatur nomine ocurreret multavit
  - subitem 3 - Lorem ipsum dolor sit amet, consectetur adipisicing elit. Malit profecta versatur nomine ocurreret multavitsubitem 3 - Lorem ipsum dolor sit amet, consectetur adipisicing elit. Malit profecta versatur nomine ocurreret multavitsubitem 3 - Lorem ipsum dolor sit amet, consectetur adipisicing elit. Malit profecta versatur nomine ocurreret multavitsubitem 3 - Lorem ipsum dolor sit amet, consectetur adipisicing elit. Malit profecta versatur nomine ocurreret multavitsubitem 3 - Lorem ipsum dolor sit amet, consectetur adipisicing elit. Malit profecta versatur nomine ocurreret multavitsubitem 3 - Lorem ipsum dolor sit amet, consectetur adipisicing elit. Malit profecta versatur nomine ocurreret multavitsubitem 3 - Lorem ipsum dolor sit amet, consectetur adipisicing elit. Malit profecta versatur nomine ocurreret multavitsubitem 3 - Lorem ipsum dolor sit amet, consectetur adipisicing elit. Malit profecta versatur nomine ocurreret multavit
  - subitem 3 - Lorem ipsum dolor sit amet, consectetur adipisicing elit. Malit profecta versatur nomine ocurreret multavit
  - subitem 3 - Lorem ipsum dolor sit amet, consectetur adipisicing elit. Malit profecta versatur nomine ocurreret multavit
  - subitem 3 - Lorem ipsum dolor sit amet, consectetur adipisicing elit. Malit profecta versatur nomine ocurreret multavit
  - subitem 3 - Lorem ipsum dolor sit amet, consectetur adipisicing elit. Malit profecta versatur nomine ocurreret multavit
  - subitem 4
  - subitem 5

- item 3
  second line of item3

Lists inside columns

- item 1
- Lorem ipsum dolor sit amet, consectetur adipisicing elit. Malit profecta versatur nomine ocurreret multavit

- item 1
- Lorem ipsum dolor sit amet, consectetur adipisicing elit. Malit profecta versatur nomine ocurreret multavit

Unordered list

- item 1
- item 2
- item 3

Unordered list with longer lines

- item 1
- Lorem ipsum dolor sit amet, consectetur adipisicing elit. Malit profecta versatur nomine ocurreret multavit
- item 3

Nested lists

- item 1
- Lorem ipsum dolor sit amet, consectetur adipisicing elit. Malit profecta versatur nomine ocurreret multavit
  - subitem 1
  - subitem 2
  - subitem 3 - Lorem ipsum dolor sit amet, consectetur adipisicing elit. Malit profecta versatur nomine ocurreret multavit
  - subitem 3 - Lorem ipsum dolor sit amet, consectetur adipisicing elit. Malit profecta versatur nomine ocurreret multavit
  - subitem 3 - Lorem ipsum dolor sit amet, consectetur adipisicing elit. Malit profecta versatur nomine ocurreret multavit
  - subitem 3 - Lorem ipsum dolor sit amet, consectetur adipisicing elit. Malit profecta versatur nomine ocurreret multavitsubitem 3 - Lorem ipsum dolor sit amet, consectetur adipisicing elit. Malit profecta versatur nomine ocurreret multavitsubitem 3 - Lorem ipsum dolor sit amet, consectetur adipisicing elit. Malit profecta versatur nomine ocurreret multavitsubitem 3 - Lorem ipsum dolor sit amet, consectetur adipisicing elit. Malit profecta versatur nomine ocurreret multavitsubitem 3 - Lorem ipsum dolor sit amet, consectetur adipisicing elit. Malit profecta versatur nomine ocurreret multavitsubitem 3 - Lorem ipsum dolor sit amet, consectetur adipisicing elit. Malit profecta versatur nomine ocurreret multavitsubitem 3 - Lorem ipsum dolor sit amet, consectetur adipisicing elit. Malit profecta versatur nomine ocurreret multavitsubitem 3 - Lorem ipsum dolor sit amet, consectetur adipisicing elit. Malit profecta versatur nomine ocurreret multavit

  - subitem 3 - Lorem ipsum dolor sit amet, consectetur adipisicing elit. Malit profecta versatur nomine ocurreret multavit
  - subitem 3 - Lorem ipsum dolor sit amet, consectetur adipisicing elit. Malit profecta versatur nomine ocurreret multavit
  - subitem 3 - Lorem ipsum dolor sit amet, consectetur adipisicing elit. Malit profecta versatur nomine ocurreret multavit
  - subitem 3 - Lorem ipsum dolor sit amet, consectetur adipisicing elit. Malit profecta versatur nomine ocurreret multavit
  - subitem 4
  - subitem 5
- item 3
  second line of item3

Lists inside columns

- item 1
- Lorem ipsum dolor sit amet, consectetur adipisicing elit. Malit profecta versatur nomine ocurreret multavit

- item 1
- Lorem ipsum dolor sit amet, consectetur adipisicing elit. Malit profecta versatur nomine ocurreret multavit

Unordered list

- item 1
- item 2
- item 3

Unordered list with longer lines

- item 1
- Lorem ipsum dolor sit amet, consectetur adipisicing elit. Malit profecta versatur nomine ocurreret multavit
- item 3

Nested lists

- item 1
- Lorem ipsum dolor sit amet, consectetur adipisicing elit. Malit profecta versatur nomine ocurreret multavit
  - subitem 1
  - subitem 2
  - subitem 3 - Lorem ipsum dolor sit amet, consectetur adipisicing elit. Malit profecta versatur nomine ocurreret multavit
  - subitem 3 - Lorem ipsum dolor sit amet, consectetur adipisicing elit. Malit profecta versatur nomine ocurreret multavit
  - subitem 3 - Lorem ipsum dolor sit amet, consectetur adipisicing elit. Malit profecta versatur nomine ocurreret multavit
  - subitem 3 - Lorem ipsum dolor sit amet, consectetur adipisicing elit. Malit profecta

versatur nomine ocurreret multavitsubitem 3 - Lorem ipsum dolor sit amet, consectetur adipisicing elit. Malit profecta versatur nomine ocurreret multavitsubitem 3 - Lorem ipsum dolor sit amet, consectetur adipisicing elit. Malit profecta versatur nomine ocurreret multavitsubitem 3 - Lorem ipsum dolor sit amet, consectetur adipisicing elit. Malit profecta versatur nomine ocurreret multavitsubitem 3 - Lorem ipsum dolor sit amet, consectetur adipisicing elit. Malit profecta versatur nomine ocurreret multavitsubitem 3 - Lorem ipsum dolor sit amet, consectetur adipisicing elit. Malit profecta versatur nomine ocurreret multavitsubitem 3 - Lorem ipsum dolor sit amet, consectetur adipisicing elit. Malit profecta versatur nomine ocurreret multavitsubitem 3 - Lorem ipsum dolor sit amet, consectetur adipisicing elit. Malit profecta versatur nomine ocurreret multavit

    - subitem 3 - Lorem ipsum dolor sit amet, consectetur adipisicing elit. Malit profecta versatur nomine ocurreret multavit
    - subitem 3 - Lorem ipsum dolor sit amet, consectetur adipisicing elit. Malit profecta versatur nomine ocurreret multavit
    - subitem 3 - Lorem ipsum dolor sit amet, consectetur adipisicing elit. Malit profecta versatur nomine ocurreret multavit
    - subitem 3 - Lorem ipsum dolor sit amet, consectetur adipisicing elit. Malit profecta versatur nomine ocurreret multavit
    - subitem 4
    - subitem 5
- item 3
  second line of item3

Lists inside columns

- item 1
- Lorem ipsum dolor sit amet, consectetur adipisicing elit. Malit profecta versatur nomine ocurreret multavit

- item 1
- Lorem ipsum dolor sit amet, consectetur adipisicing elit. Malit profecta versatur nomine ocurreret multavit

Unordered list

- item 1
- item 2
- item 3

Unordered list with longer lines

- item 1
- Lorem ipsum dolor sit amet, consectetur adipisicing elit. Malit profecta versatur nomine ocurreret multavit
- item 3

Nested lists

- item 1
- Lorem ipsum dolor sit amet, consectetur adipisicing elit. Malit profecta versatur nomine

ocurreret multavit

- subitem 1
- subitem 2
- subitem 3 - Lorem ipsum dolor sit amet, consectetur adipisicing elit. Malit profecta versatur nomine ocurreret multavit
- subitem 3 - Lorem ipsum dolor sit amet, consectetur adipisicing elit. Malit profecta versatur nomine ocurreret multavit
- subitem 3 - Lorem ipsum dolor sit amet, consectetur adipisicing elit. Malit profecta versatur nomine ocurreret multavit
- subitem 3 - Lorem ipsum dolor sit amet, consectetur adipisicing elit. Malit profecta versatur nomine ocurreret multavitsubitem 3 - Lorem ipsum dolor sit amet, consectetur adipisicing elit. Malit profecta versatur nomine ocurreret multavitsubitem 3 - Lorem ipsum dolor sit amet, consectetur adipisicing elit. Malit profecta versatur nomine ocurreret multavitsubitem 3 - Lorem ipsum dolor sit amet, consectetur adipisicing elit. Malit profecta versatur nomine ocurreret multavitsubitem 3 - Lorem ipsum dolor sit amet, consectetur adipisicing elit. Malit profecta versatur nomine ocurreret multavitsubitem 3 - Lorem ipsum dolor sit amet, consectetur adipisicing elit. Malit profecta versatur nomine ocurreret multavitsubitem 3 - Lorem ipsum dolor sit amet, consectetur adipisicing elit. Malit profecta versatur nomine ocurreret multavitsubitem 3 - Lorem ipsum dolor sit amet, consectetur adipisicing elit. Malit profecta versatur nomine ocurreret multavit
- subitem 3 - Lorem ipsum dolor sit amet, consectetur adipisicing elit. Malit profecta versatur nomine ocurreret multavit
- subitem 3 - Lorem ipsum dolor sit amet, consectetur adipisicing elit. Malit profecta versatur nomine ocurreret multavit
- subitem 3 - Lorem ipsum dolor sit amet, consectetur adipisicing elit. Malit profecta versatur nomine ocurreret multavit
- subitem 3 - Lorem ipsum dolor sit amet, consectetur adipisicing elit. Malit profecta versatur nomine ocurreret multavit
- subitem 4
- subitem 5

- item 3
  second line of item3
